# Relationship between serum MMP-9 level and prognosis after radical resection for Hilar cholangiocarcinoma patients^1^

**DOI:** 10.1590/s0102-865020190040000009

**Published:** 2019-04-29

**Authors:** Ou Li, Weimin Yi, Pingzhou Yang, Chao Guo, Chuang Peng

**Affiliations:** I MD, Department of Hepatobiliary Surgery, Hunan Provincial People’s Hospital, People’s Republic of China. Technical procedures, manuscript writing.; II MD, Department of Hepatobiliary Surgery, Hunan Provincial People’s Hospital, People’s Republic of China. Technical procedures, analysis of data.; III MD, Department of Hepatobiliary Surgery, Hunan Provincial People’s Hospital, People’s Republic of China. Design of the study, critical revision.

**Keywords:** Klatskin Tumor, Matrix Metalloproteinase 9, Postoperative Period, Prognosis

## Abstract

**Purpose:**

To analyze the preoperative serum matrix metalloproteinase-9 (MMP-9) levels and prognosis of patients with hilar cholangiocarcinoma (HC) undergoing radical resection.

**Methods:**

Preoperative serum MMP-9 levels in patients with HC undergoing radical resection were detected by enzyme-linked immunosorbent assay (ELISA). The ROC curve assay was used to analyze the preoperative serum MMP-9 level to determine the most valuable cut-off point. The relationship between MMP-9 and clinicopathological features of HC patients was analyzed. Kaplan-Meier method was used to analyze the prognostic factors, and COX regression model was used to analyze the independent risk factors affecting prognosis.

**Results:**

Preoperative serum MMP-9 levels were significantly elevated in the death patients compared with the survival patients. The most valuable cut-off point for preoperative serum MMP-9 for prognosis was 201.93 ng/mL. Preoperative serum MMP-9 was associated with Bismuth-Corlette classification) and lymph node metastasis. Kaplan-Meier analysis showed that MMP-9, Bismuth-Corlette classification, Lymph node metastasis, Portal vein invasion, Hepatic artery invasion, Liver invasion, Incised margin, and Preoperative biliary drainage were related to prognosis. Cox regression model confirmed that hepatic artery invasion, liver invasion, incised margin, and MMP-9 have the potential to independence predicate prognosis in HC patients.

**Conclusion:**

Preoperative serum MMP-9 has high predictive value for prognosis and is an independent influencing factor for the prognosis of patients with hilar cholangiocarcinoma.

## Introduction

 Hilar cholangiocarcinoma (HC), also known as Klatskin tumor, is a malignant tumor that grows in the confluence of the left and right hepatic ducts and the common hepatic duct. In cholangiocarcinoma, HC is more common that is accounting for about 58%-66%^1^. HC has a high mortality rate in the early stages and is considered to be one of the most deadly malignant tumors^2^. However, the cause of HC is not yet clear. Liver resection and liver transplantation in HC patients are currently the only radical surgery^3^, however, only a small number of patients still have the opportunity to undergo radical surgery after diagnosis, and the postoperative prognosis is poor. Other adjuvant therapies such as chemotherapy and radiotherapy have not achieved satisfactory results in improving the prognosis of patients^4^. Studying factors related to prognosis after surgical treatment and finding related markers for preventing HC recurrence or metastasis are of great significance for improving the long-term survival of HC patients.

 Matrix metalloproteinase-9 (MMP-9), also known as gelatinase B or type IV collagenase B, is a zinc-dependent proteolytic enzyme involved in the degradation of many different components of the basement membrane and subsequent remodeling of extracellular matrix (ECM)^5^. MMP-9 not only plays a key role in the development of cancers, but also their characteristics of being secreted into the blood stream have inspired many researchers to evaluate the associations between circulating level of MMP-9 and clinicopathological characteristics of cancers^6^. Clinical studies have shown that high expression of MMP-9 is associated with clinical pathological features of cancer such as lymph node metastasis and tumor differentiation^7^. Elevated serum MMP-9 often suggests a poor prognosis^8^. However, there are few studies on the relationship between MMP-9 and the histopathological features and prognosis of HC. There is currently no correlation report on the preoperative serum MMP-9 level predicting the prognosis of HC.

 In this study, patients with HC radical surgery were enrolled. Enzyme-linked immunosorbent assay (ELISA) was used to analyze the most valuable cut-off point of preoperative serum MMP-9 for the prognosis of HC radical resection, and to explore the influencing factors of prognosis of HC radical mastectomy. We hope to provide appropriate targets for prognosis monitoring after HC.

## Methods

 This study was approved by the Ethics Committee of the Hunan Provincial People’s Hospital.

**Figure 1:**
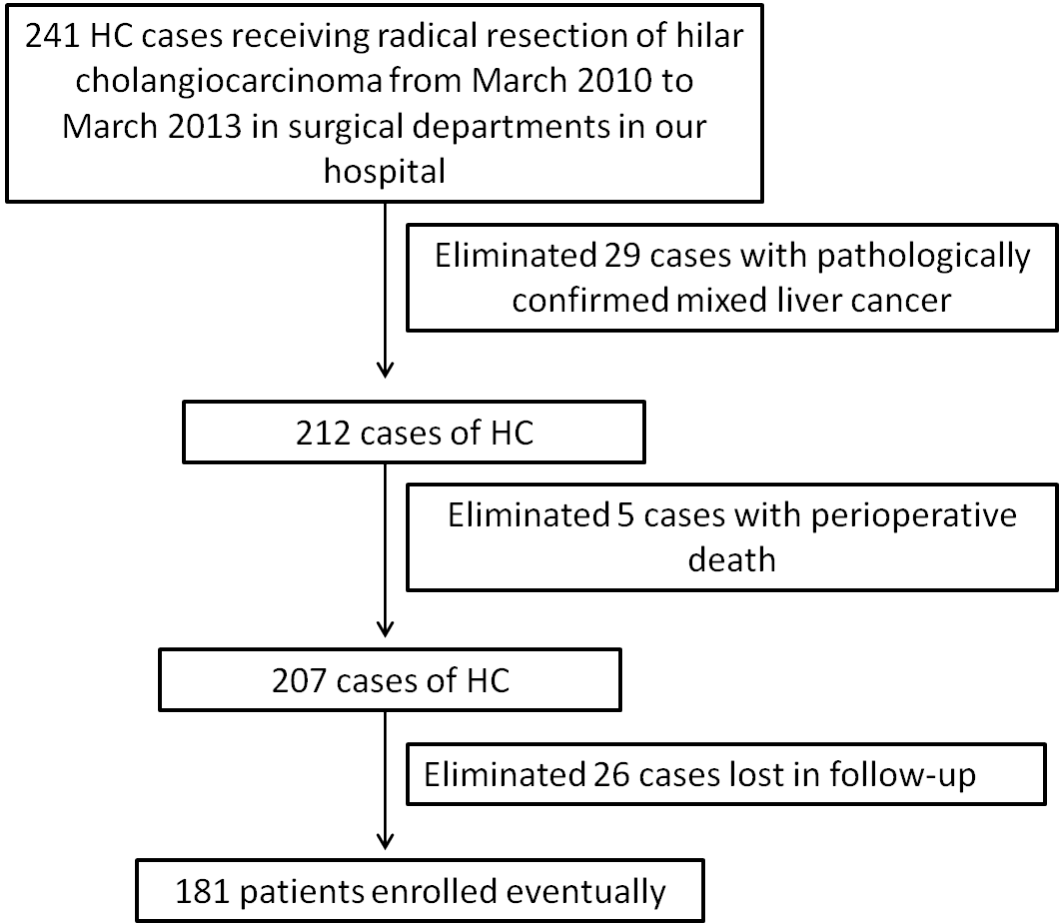
Inclusion criteria and censored data of the patients.

 Two hundred and forty-one patients with HC who were admitted to Hunan Provincial People’s Hospital from March 2010 to March 2013 were included in the study. All patients underwent radical surgery and were diagnosed by pathology after surgery. Patients with perioperative death were excluded. Pathological results were excluded from mixed liver cancer, as well as patients who were lost to follow-up. The causes of death in follow-up cases were surgical factors (non-other cases, such as heart disease). Finally, 181 eligible cases were included, including 103 males and 78 females, aged 35-78 years. All patients were informed of the relevant conditions of the study and the required indicators to be recorded before surgery. Patients were signed informed consent and followed up regularly. The deadline for follow-up is June 30, 2018. The overall survival (OS) time is from the date of surgery to the date of death or the date of follow-up. All experiments were performed at Department of Hepatobiliary Surgery, Hunan Provincial People’s Hospital, China. Patient enrollment and follow-up are shown in Figure 1.

### Detection of serum MMP-9 content

 Three milliliters of venous peripheral blood of HC patients was collected before radical surgery, and centrifuged to obtain serum, which was then stored at -80°C. The concentration of MMP-9 was measured by ELISA. ELISA kit for MMP-9 WAS purchased from R&D Systems Inc. Assays were performed according to the manufacturer’s instructions.

### Statistical analyses

 Analysis was performed using SPSS 17.0 software (SPSS Inc, Chicago, IL). Measurement data conforming to the normal distribution were expressed as mean ± standard deviation(mean ± SD), Student t test was used for comparing the average of two groups. The predictive efficacy of serum MMP-9 on prognosis was assessed using the receiver operating characteristic curve (ROC).The most prognostic value of serum MMP-9 was calculated using the most approximate index. Count data was expressed in terms of number of cases (n), using χ 2 test or Fisher exact probability method to analyze. The survival curve was drawn using the Kaplan-Meier method. Differences in survival curves were detected by Log-rank method. Cox regression model was established, and the significance level of the variables retained by the model: the entry criterion was *p*<0.05. At the α=0.05 test level, the Backward LR method was used to screen out the independent risk factors affecting the prognosis of HC patients. The degree of influence of risk factors is expressed as the hazard ratio (HR). If *p*< 0.05 then the difference was considered statistically significant.

## Results

### Follow-up

**Figure 2:**
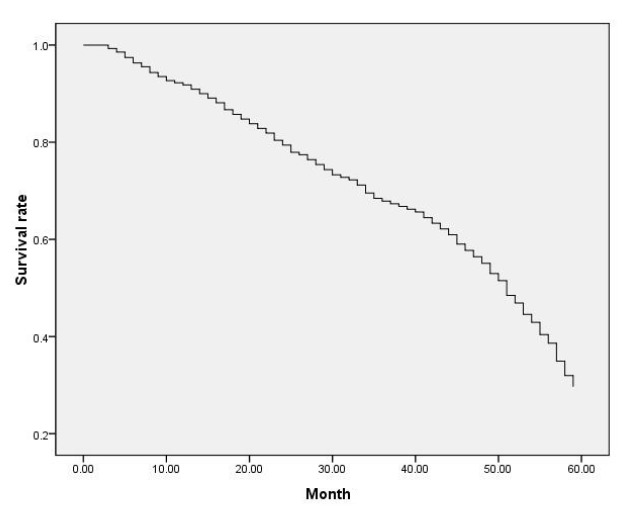
Overall survival curve of HC patients after surgery.

 To the follow-up deadline, 120 of the 181 patients died, with a minimum survival time of 3 months, a maximum of 59 months, and a median survival of 46 months. The overall survival curve of the patient is shown in Figure 2. The 1-, 3-, and 5-year survival rates were 88.4% (160/181), 73.5% (133/181), and 33.7% (61/181), respectively.

 The relationship between preoperative serum MMP-9 level and prognosis of HC patients

**Figure 3:**
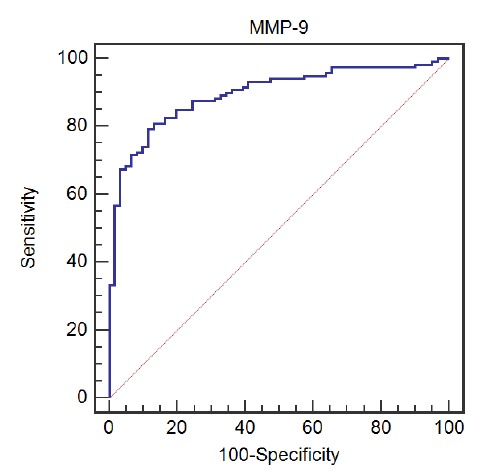
Preoperative serum MMP-9 predicts ROC curve analysis of postoperative death in HC patients.

 The preoperative serum MMP-9 concentration in 181 patients with HC was (216.7±72.1) ng/mL. Preoperative serum MMP-9 levels were significantly elevated in the death group compared with the survival group (155.50±41.79 *vs.* 251.57±62.02, *p*<0.001). To determine the most valuable MMP-9 level cut-off point for prognosis, we applied the ROC curve method for analysis. We found that the area under the ROC curve of serum MMP-9 levels predicting patient mortality was 0.896 (Fig. 3), confirming that preoperative serum MMP-9 has a higher value in predicting postoperative prognosis in HC patients. When the MMP-9 level was 201.93 ng/mL, the Youden index was the largest. The sensitivity of MMP-9≥201.93 ng/mL for predicting death in HC patients was 80.33%, and the specificity was 86.89%. The result hinted that preoperative serum MMP-9 levels may be potential markers for predicting the prognosis of HC patients.

### Relationship between serum MMP-9 level and clinicopathological features of HC patients

 To analyze the relationship between preoperative serum MMP-9 and clinicopathological features of HC patients, we classified the variables related to clinicopathological features according to their own characteristics. As shown in Table 1, preoperative serum MMP-9 levels were not associated with age, sex, tumor size, differentiation, nerve invasion, portal vein invasion, hepatic artery invasion, liver invasion, incised margin, total bilirubin, preoperative biliary drainage and postoperative radiotherapy, but were associated with Bismuth-Corlette classification (χ2=9.442, *p*=0.002) and lymph node metastasis (χ2=9.026, *p*=0.003).

**Table 1:** Relationship between serum MMP-9 level and clinicopathological features of HC patients (n, %).

Item	n	MMP-9 (ng/mL)			p
		<201.93 (n=75)		≥201.93 (n=106)	
Age	<60 years	97	38(50.7%)	59(55.7%)	0.507
	≥60 years	84	37(49.3%)	47(44.3%)	
Sex	Male	103	42(56.0%)	61(57.6%)	0.836
	Female	78	33(44.0%)	45(42.4%)	
Tumor size	<2.5 cm	113	51(68.0%)	62(58.5%)	0.193
	≥2.5 cm	68	24(32.0%)	44(41.5%)	
Differentiation	High and moderate	174	72(96.0%)	102(96.2%)	0.754
	Poor	7	3(4.0%)	4(3.8%)	
Bismuth-Corlette classification	I+II	42	26(34.7%)	16(15.1%)	0.002
	IIIa+IIIb+IV	139	49(65.3%)	90(84.9%)	
Lymph node metastasis	Negative	117	58(77.3%)	59(55.7%)	0.003
	Positive	64	17(22.7%)	47(44.3%)	
Nerve invasion	Negative	109	49(65.3%)	60(56.6%)	0.237
	Positive	72	26(34.7%)	46(43.4%)	
Portal vein invasion	Negative	126	58(77.3%)	68(64.2%)	0.057
	Positive	55	17(22.7%)	38(35.8%)	
Hepatic artery invasion	Negative	142	56(74.7%)	86(81.1%)	0.297
	Positive	39	19(25.3%)	20(18.9%)	
Liver invasion	Negative	132	56(74.7%)	76(71.7%)	0.658
	Positive	49	19(25.3%)	30(28.3%)	
Incised margin	Negative(R0)	151	66(88.0%)	85(80.2%)	0.164
	Positive(R1)	30	9(12.0%)	21(19.8%)	
Total bilirubin	<100 mg/L	77	35(46.7%)	42(39.6%)	0.345
	≥100 mg/L	104	40(53.3%)	64(60.4%)	
Preoperative biliary drainage	Undone	126	54(72.0%)	72(67.9%)	0.557
	Done	55	21(28.0%)	34(32.1%)	
Postoperative radiotherapy	Undone	109	47(62.7%)	62(54.5%)	0.572
	Done	72	28(37.3%)	44(41.5%)	

### High MMP-9 levels are correlated with poor prognosis in HC patients

**Figure 4:**
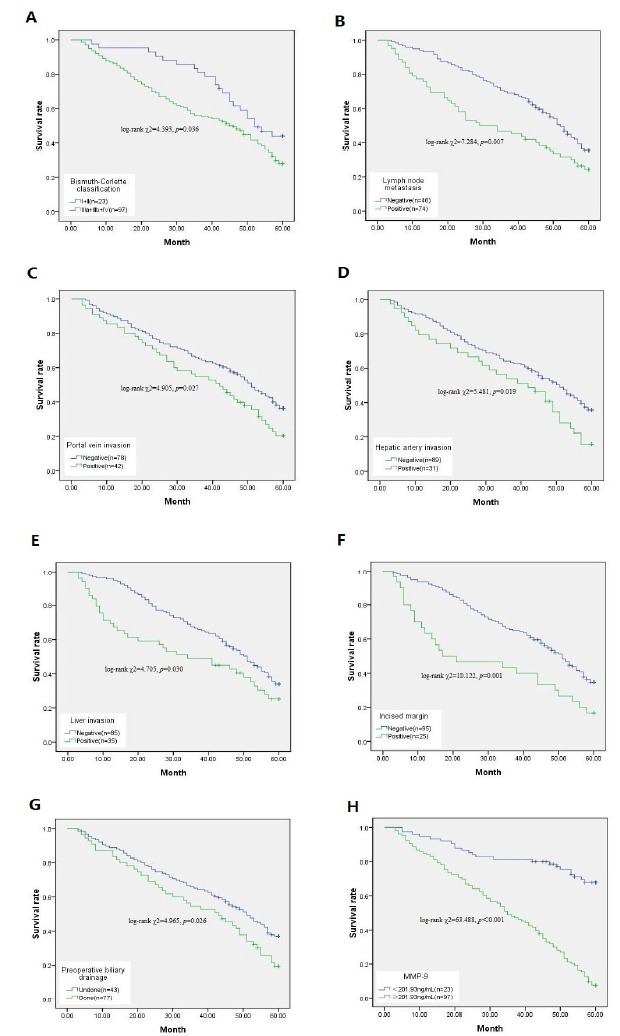
Relationship between clinical pathological parameters and preoperative serum MMP-9 and postoperative survival of HC patients. **A**: Bismuth-Corlette classification. **B**: Lymph node metastasis. **C**: Portal vein invasion. **D**: Hepatic artery invasion. **E**: Liver invasion. **F**: Incised margin. **G**: Preoperative biliary drainage. **H**: Preoperative serum MMP-9. “n” represents the number of deaths.

 To explore whether clinical pathological parameters and preoperative serum MMP-9 levels were associated with OS, we utilized Kaplan Meier survival analysis and log-rank test (Table 2, Fig. 4). The median survival time was 34.5 months in patients with high MMP-9 (MMP-9≥201.93) and 50.9 months in patients with low MMP-9 (MMP-9<201.93 ng/mL). The Kaplan-Meier results showed (Table 2, Fig. 4H) that patients with high MMP-9 had a significantly lower median survival time than patients with low MMP-9 (log-rank χ2=63.488, *p*<0.001). Subsequently, Bismuth-Corlette classification (log-rank χ2=4.393, *p*=0.036), Lymph node metastasis (log-rank χ2=7.284, *p*=0.007), Portal vein invasion (log-rank χ2=4.905, *p*=0.027), Hepatic artery invasion (log-rank χ2=5.481, *p*=0.019), Liver invasion (log-rank χ2=4.705, *p*=0.030), Incised margin (log-rank χ2=10.122, *p*=0.001), and Preoperative biliary drainage (log-rank χ2=4.965, *p*=0.026) were also related to prognosis (Table 2, Fig. 4A-G,).

**Table 2:** Univariate analysis of potential predictors for prognosis of HC patients.

Item	n	Median of survival time (month)		OS rate(%)	p
Age	<60 years	97	41.1	34.0%	0.983
	≥60 years	84	41.3	33.3%	
Sex	Male	103	41.0	34.0%	0.953
	Female	78	41.5	33.3%	
Tumor size	<2.5 cm	114	42.4	36.0%	0.320
	≥2.5 cm	67	39.3	29.9%	
Differentiation	High and moderate	174	42.1	33.9%	0.060
	Poor	7	20.7	28.6%	
Bismuth-Corlette classification	I+II	42	48.2	45.2%	0.036
	IIIa+IIIb+IV	139	39.1	30.2%	
Lymph node metastasis	Negative	117	45.0	37.8%	0.007
	Positive	64	33.9	25.8%	
Nerve invasion	Negative	109	42.3	38.5%	0.134
	Positive	72	39.6	26.4%	
Portal vein invasion	Negative	126	42.9	38.1%	0.027
	Positive	55	37.4	23.6%	
Hepatic artery invasion	Negative	142	42.5	37.3%	0.019
	Positive	39	36.4	20.5%	
Liver invasion	Negative	132	44.0	35.6%	0.030
	Positive	49	33.8	28.6%	
Incised margin	Negative(R0)	151	43.5	37.1%	0.001
	Positive(R1)	30	29.6	16.7%	
Total bilirubin	<100 mg/L	77	40.7	35.1%	0.868
	≥100 mg/L	104	41.6	32.7%	
Preoperative biliary drainage	Undone	126	42.8	38.9%	0.026
	Done	55	37.6	21.8%	
Postoperative radiotherapy	Undone	109	40.9	33.0%	0.825
	Done	72	41.8	34.7%	
MMP-9	<201.93 ng/mL	75	50.9	69.3%	<0.001
	≥201.93 ng/mL	106	34.5	8.5%	

Note: OS: overall survival; MMP-9: matrix metalloproteinase-9.

### Cox regression model

 Next, we included the statistically significant differences in the results of the univariate analysis into the Cox regression model for multivariate analysis. Multivariate analysis confirmed that hepatic artery invasion (*p*=0.016), liver invasion (*p*=0.044), incised margin (*p*=0.012), and MMP-9 (*p*<0.001) have the potential to independently predicate prognosis in HC patients (Table 3). 

**Table 3:** Multivariate analysis of potential predictors for prognosis of HC patients.

Variable	HR	Chi-square	***p* value**
Lymph node metastasis	1.430	3.388	0.066
Hepatic artery invasion	1.710	5.766	0.016
Liver invasion	1.530	4.055	0.044
Incised margin	1.849	6.335	0.012
Preoperative serum MMP-9	5.192	61.467	<0.001

Note: MMP-9: matrix metalloproteinase-9; HR: high-risk ratio.

## Discussion

 MMPs are one of the most important family of proteolytic enzymes that play an important role in embryonic development, differentiation, tumor angiogenesis, tumor invasion and metastasis^9^. It has been demonstrated that MMPs-mediated degradation of extracellular matrix can lead to invasion and metastasis of tumor cells^10^ ^,^ ^11^, and abnormal expression of MMPs has prognostic significance in some human malignant tumors^12^ ^,^ ^13^. Deng *et al.* ^14^ found that MMP-2 protein levels were significantly upregulated in colorectal cancer (CRC), and MMP-2 expression could be novel diagnostic and prognostic markers for CRC patients. Yao *et al*.^15^ revealed that MMP-2 and MMP-9 expression were both independent factors of prognosis and lymphnode metastasis in patients with early gastric cancer. A meta-analysis also showed a significant poor prognostic effect of MMP-7 in gastric cancer survival^16^. MMP-9 is more well know in the MMPs family, which can degrade type IV collagen in ECM, allowing cancer cells to break through the basement membrane of the primary site^17^. MMP-9 can attenuate the basement membrane of blood vessels and lymphatic vessels, allowing cancer cells to infiltrate directly into the vasculature^18^ to participate in tumor metastasis and invasion^19^. Some scholars have conducted immunohistochemistry to confirm that MMP-9 has a high value in predicting the long-term prognosis of multiple tumors^20^ ^,^ ^21^. Zhao *et al*.^22^ used immunohistochemistry to analyze the expression of MMP-9 in tumor tissues of 127 patients with tirple-negative breast cancer, and found that patients with high MMP-9 expression had poor prognosis. Interestingly, the results of some scholars have been relatively novel, which confirms that MMP-9 may be a protective factor in the carcinogenesis and metastasis of tumors. For example, MMP-9 expression is reduced in some head and neck malignancies with local metastasis^23^ and elevated MMP-9 expression may indicate better overall survival in salivary adenocarcinoma^24^. Even more surprising is that in breast cancer^25^ and enteritis-related cancers^26^, MMP-9 expression also indicates a relatively good prognosis. These results suggest that MMP-9 can act as both a tumor-promoting factor and a protective factor, and its relationship with disease prognosis may depend on the specific environment of action.

 Sun *et al.* ^27^ found that MMP-9 overexpression was observed in tumor tissues of 46.5% of patients with cholangiocarcinoma of the liver. Their study also found that although MMP-9 overexpression was not associated with patient clinicopathological parameters, overall survival was significantly lower in patients with high MMP-9 expression than in patients with negative or low MMP-9 expression, suggesting that the expression of MMP-9 in tissues is of great significance in the evaluation of postoperative prognosis of intrahepatic cholangiocarcinoma. Recent studies have shown that real-time monitoring of non-invasive serum biomarkers targeting prognosis has greater safety and efficacy^28^. MMP-9 levels in the peripheral circulation have also been shown to be associated with metastasis and long-term prognosis in a variety of cancers. Sung *et al*.^29^ tested the preoperative serum MMP-9 levels in breast cancer patients and found that elevated MMP-9 levels were associated with decreased breast cancer survival rate. Lin *et al*.^30^ also reported that plasma MMP-9 was significantly elevated in thyroid cancer patients with lymph node invasion and distant metastasis, and suggested that regular detection of plasma MMP-9 may help to determine the distant metastasis tendency. However, the relationship between preoperative serum MMP-9 levels and postoperative prognosis in HC patients has not been reported. Therefore, we focused on serum MMP-9 levels and studied the relationship between preoperative serum MMP-9 levels and postoperative prognosis in HC patients.

 We used the ROC curve method to analyze the MMP-9 cut-off point, which has the most prognostic value after HC radical surgery. The results showed that the area under the ROC curve was the largest (AUC=0.896) when the cut-off point was 201.93 ng/mL, which was the most valuable value. Kaplan-Meier analysis confirmed that the median survival time of HC patients with low serum MMP-9 levels was significantly higher than that of patients with high serum MMP-9 levels. These results confirm that preoperative serum MMP-9 can be used as a useful marker to predict postoperative prognosis in HC patients. Furthermore, we found that preoperative serum MMP-9 levels were associated with Bismuth-Corlette classification and Lymph node metastasis, which may be related to the lower sensitivity of MMP-9 in predicting the prognosis of HC patients. Our results suggested that MMP-9 may be involved in the progression and metastasis of HC disease, which may be related to the potential carcinogenesis of MMP-9, but the related mechanism remains to be further explored. We further included statistically significant clinical pathological factors and preoperative serum MMP-9 levels in the multivariate survival analysis model. It was found that high serum MMP-9 levels is an independent risk factor for predicting the prognosis of HC patients. Additionally, Cox analysis showed that risk factors associated with prognosis of patients with HC include portal vein invasion, hepatic artery invasion, liver invasion, preoperative biliary drainage, etc., which may also affect the sensitivity of MMP-9 by affecting the immune response state of the body. 

## Conclusions

 As far as we know, this is the only study to evaluate the relationship between preoperative serum MMP-9 levels and postoperative prognosis in HC patients. In this study, we showed that high MMP-9 levels have a high predictive value for postoperative prognosis in patients with HC undergoing radical resection, and are one of the independent factors influencing postoperative prognosis. Therefore, evaluation of serum MMP-9 levels may be helpful in guiding the prognosis of HC patients and subsequent treatment options. However, more studies are necessary to confirm our data.
